# High‐dose hydroxocobalamin achieves biochemical correction and improvement of neuropsychiatric deficits in adults with late onset cobalamin C deficiency

**DOI:** 10.1002/jmd2.12087

**Published:** 2019-12-13

**Authors:** Tomoyasu Higashimoto, Alexander Y. Kim, Jessica T. Ogawa, Jennifer L. Sloan, Mohammed A. Almuqbil, Julia M. Carlson, Irini Manoli, Charles P. Venditti, Meral Gunay‐Aygun, Tao Wang

**Affiliations:** ^1^ Department of Genetic Medicine and Pediatrics Johns Hopkins University Baltimore Maryland; ^2^ Medical Genomics and Metabolic Genetics Branch National Institute of Human Genome Research, National Institutes of Health Bethesda Maryland; ^3^ Division of Pediatric Neurology King Saud bin Abdulaziz University for Health Sciences Riyadh Saudi Arabia; ^4^ King Abdullah International Medical Research Center King Abdullah Specialist Children's Hospital – Ministry of National Guard Riyadh Saudi Arabia; ^5^ Department of Neurology Johns Hopkins University Baltimore Maryland

**Keywords:** *cblC*, cobalamin C, combined methylmalonic acidemia and homocystinuria, Hydroxocobalamin, intracellular cobalamin metabolism, MMACHC

## Abstract

Cobalamin C (*cblC*) deficiency is the most common inborn error of intracellular cobalamin metabolism caused by pathogenic variant(s) in *MMACHC* and manifests with methylmalonic acidemia, hyperhomocysteinemia, and hypomethioninemia with a variable age of presentation. Individuals with late‐onset *cblC* may be asymptomatic until manifesting neuropsychiatric symptoms, thromboembolic events, and renal disease. Although hydroxocobalamin provides a foundation for therapy, optimal dose regimen for adult patients has not been systematically evaluated. We report three adult siblings with late‐onset *cblC* disease, and their biochemical and clinical responses to high‐dose hydroxocobalamin. The 28‐year‐old proband presented with severe psychosis, progressive neurological deterioration, and deep venous thrombosis complicated by a pulmonary embolism. MRI studies identified lesions in the spinal cord, periventricular white matter, and basal ganglia. Serum homocysteine and methylmalonic acid levels were markedly elevated. Hydroxocobalamin at standard dose (1 mg/day) initially resulted in partial metabolic correction. A regimen of high‐dose hydroxocobalamin (25 mg/day) together with betaine and folic acid resulted in rapid and sustainable biochemical correction, resolution of psychosis, improvement of neurological functions, and amelioration of brain and spinal cord lesions. Two siblings who did not manifest neuropsychiatric symptoms or thromboembolism achieved a satisfactory metabolic control with the same high‐dose regimen. Hydroxocobalamin injection was then spaced out to 25 mg weekly with good and sustainable metabolic control. All three patients are compound heterozygotes for c.271dupA p.Arg91LysfsX14 and c.389A > G p.Tyr130Cys. This study highlights the importance of evaluating intracellular cobalamin metabolism in adults with neuropsychiatric manifestations and/or thromboembolic events, and demonstrates that high‐dose hydroxocobalamin achieves rapid and sustainable metabolic control and improvement in neuropsychiatric outcomes in adults with late‐onset *cblC* disease.

**SYNOPSIS**

High‐dose hydroxocobalamin achieves rapid and sustainable biochemical correction and improvement of neuropsychiatric deficits in adults with *cblC* deficiency.

## 1 INTRODUCTION

Cobalamin C deficiency (*cblC*; OMIM: #277400) is the most common inborn error of intracellular cobalamin metabolism and most often caused by pathogenic variants in *MMACHC* (methylmalonic aciduria and homocystinuria type C).^1^, ^2^, ^3^ MMACHC deficiency impairs the conversion of vitamin B12 into the two metabolically active cobalamins, adenosylcobalamin (AdoCbl) and methylcobalamin (MeCbl). AdoCbl is a cofactor for methylmalonyl‐CoA mutase, which converts methylmalonyl‐CoA into succinyl‐CoA in the mitochondria. MeCbl is a cofactor for methionine synthase, which converts homocysteine into methionine in the cytosol.^1^, ^2^, ^4^ Biochemically, *cblC* deficiency is characterized by elevation of methylmalonic acid (MMA) and total homocysteine (tHcy), and reduction of methionine levels in serum.^1^, ^2^ Clinically, *cblC* deficiency displays variable signs and symptoms, primarily neurologic, ophthalmologic and hematologic manifestations, and historically has been classified into early‐ and late‐onset forms.^4^, ^5^ The late‐onset form presents from early childhood to adulthood with progressive neurological deterioration, severe psychiatric disturbances, cognitive impairment, chronic renal disease, hemolytic uremic syndrome, and/or thromboembolic complications.^6^, ^7^, ^8^ The variable age of onset and wide‐spectrum of clinical presentations, which often overlap with those of common diseases, pose diagnostic challenges in adults with late‐onset *cblC* deficiency.^4^, ^9^

Practice guidelines for the clinical diagnosis and management of *cblC* and related remethylation disorders have been developed.^10^ Parenteral hydroxocobalamin injection, generally 1 mg/day, is used to increase intracellular cobalamin concentration to maximize biochemical response. Betaine is used to lower homocysteine as a substrate for betaine‐homocysteine methyltransferase, which can convert homocysteine into methionine. Other supplements are also employed to improve biochemical parameters including folic/folinic acid to enhance the remethylation pathway,^4^ methionine to prevent its deficiency, and carnitine for excretion of propionyl‐CoA groups and prevent carnitine deficiency. Treatment with hydroxocobalamin has been shown to improve survival and some clinical symptoms in children with *cblC* deficiency.^6^, ^11^ However, the frequency and dosing has not been studied extensively and biochemical and clinical responses to treatment are often variable and patient‐dependent.^12^, ^13^, ^14^, ^15^ An optimal regimen of hydroxocobalamin injection for adult patients with *cblC* deficiency has not been systematically evaluated.^10^, ^15^

We report three adult full‐siblings with biochemically and molecularly confirmed late‐onset *cblC* deficiency. The 28‐year‐old proband (sibling 2) presented with severe psychiatric symptoms, neurological deterioration, thromboembolic complications, abnormal brain, and spine findings on MRIs, and elevated serum tHcy and MMA levels. The 29‐year‐old sister (sibling 1) had unexplained chronic renal disease while the 26‐year‐old sister (sibling 3) appeared clinically asymptomatic. All three patients were treated with a high‐dose hydroxocobalamin injection regimen successfully. This case series emphasizes the need to investigate intracellular cobalamin metabolism disorders in adults with unexplained neurological deterioration, neuropsychiatric symptoms, thromboembolic events, and/or renal disease. Timely implementation of a high‐dose hydroxocobalamin regimen achieved a rapid and sustainable biochemical correction, and a drastic improvement of neuropsychiatric symptoms and neuroimaging phenotypes in our patients.

## 2 CASE REPORT

**Figure 1:**
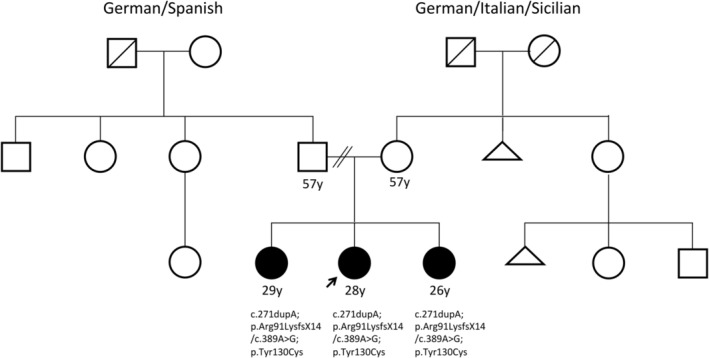
Pedigree of the proband family and genotypes of three patients with *cblC* deficiency. A three‐generation pedigree of the proband family with *cblC* deficiency was shown. Square symbol, male; circular symbol, female; symbols with an oblique line, deceased. Filled symbols, affected individuals with *cblC* deficiency. The arrow indicates the proband, sibling 2. *MMACHC* genotypes are listed below the symbol for each affected individual. Note all three affected female siblings are compound heterozygous for the same two pathogenic variants in *MMACHC*

The proband, a 28‐year‐old female and a vegetarian, had a history of migraine headaches and was otherwise in good general health. She reported a history of recent ambient, but not direct, exposure to nitrous oxide of certain quantity and an experience of flu‐like symptoms 1 month prior to developing clinical symptoms. Her family history was significant for multiple sclerosis in her mother. Her two sisters, 26 and 29 years of age, were at their baseline health as described below (Figure 1).

The proband first complained of reduced finger dexterity and gait disturbance described as “difficulty balancing.” During subsequent weeks, she developed severe paranoid delusions, blurred vision, and worsening gait. Over the next 3 months, the patient required admissions to two local hospitals for evaluation and management of acute psychosis and neurologic deterioration, including a voluntary admission to an inpatient psychiatric hospital. T2/FLAIR brain magnetic resonance imaging (MRIs) was notable for diffuse periventricular white matter changes and basal ganglia involvement. T2/FLAIR spine MRIs showed extensive dorsal white matter involvement extending from C4 to C5. Diagnostic evaluations for common infections, cerebrospinal fluid oligoclonal bands, myelin basic protein, and paraneoplastic autoantibodies were negative. Serum vitamin B12 levels were normal. She was treated with methylprednisolone for a presumed autoimmune disorder. During one hospitalization, she was diagnosed with a provoked deep venous thrombosis complicated by a pulmonary embolism. A thrombophilia evaluation was negative for abnormalities in protein C, protein S, factor V Leiden, antithrombin III, and prothrombin. She was treated with enoxaparin and apixaban.

**Figure 2:**
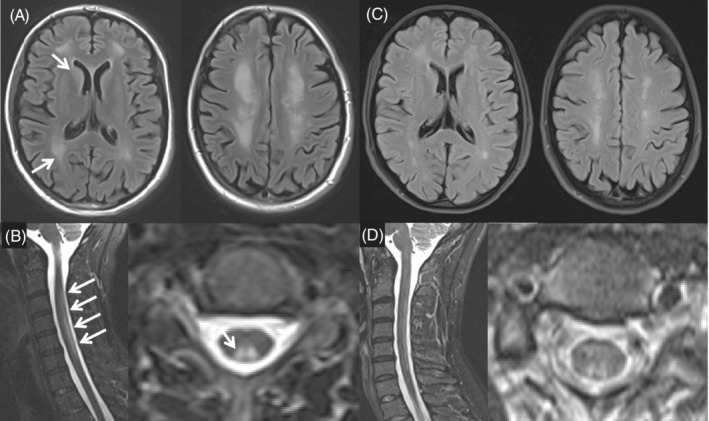
T2/FLAIR MRI Images of the brain and spinal cord of the proband patient before and after treatment. Panel A, T2/FLAIR images of the brain before treatment: note the fairly confluent hyperintensity involving periventricular white matter of the supratentorial brain. Putamen and head of caudate (arrows) are diffusely hyper‐intensive. Similar changes are shown on the medial occipitotemporal region. Panel B, T2/FLAIR images of the spinal cord before treatment: note the increase in hyperintensity in the dorsal columns from C2 to middle C6 levels and from T7 to T10 levels (arrows). No apparent loss of spinal cord volume was noted. Panel C, T2/FLAIR images of brain after treatment. Note the increased T2/FLAIR hyperintensity signals involving periventricular white matter. These lesions appear less prominent as compared to that in Panel A. The degree of increased T2/FLAIR signals involving the caudate and putamen are much less conspicuous as compared to that in panel A. Panel D, T2/FLAIR images of spinal cord after treatment. Note the increased T2 signal involving the posterior columns of the spinal cord spanning C2‐C7 levels. The foci of T2‐weighted hyperintensity signals appear much better circumscribed and smaller in cross section appearance as compared to that in panel B

The proband was admitted to our institution, 4 months after her initial presentation, for further diagnostic evaluation and management of severe progressive psychiatric symptoms and neurological deterioration. On presentation in the emergency department, she was unable to ambulate independently, developed urinary retention, had worsening paranoid delusions, severe perseverative speech, frequent panic attacks, and transient catatonia with cerea flexibilitas. Her neurologic exam was significant for poor attention, perseverative and distractible conversation, and inability to name days of the week forward or backward. Her upper extremities showed normal muscle tone, strength, and deep tendon reflexes (DTRs). Examination of her lower extremities was notable for normal strength, increased muscle tone, and increased DTRs with bilateral sustained patellar and ankle clonus with upgoing toes. Her sensory examination was remarkable for absent proprioception to her knees and absent vibratory sense to her waist, with preserved pinprick and temperature sensation. A dilated ophthalmologic examination was unremarkable. Neurologic exam localized to the dorsal columns of the spinal cord, and was concerning for diffuse cortical involvement. Multiplanar, multisequence T2/FLAIR MRIs of the brain and whole spine with and without intravenous (IV) contrast identified diffuse periventricular white matter changes, prominent basal ganglia involvement, and extensive dorsal white matter lesions extending from C2‐T10 of the spinal cord (Figure 2A,B). Her laboratory evaluations revealed a normal serum vitamin B12 (391 pg/mL; normal 232‐1245 pg/mL) and increased folic acid (>20 ng/mL; normal >4.7 ng/mL), significantly elevated serum levels of MMA (165.8 μmol/L; normal 0.045‐0.325 μmol/L) and tHcy (201.9 μmol/L; normal 0‐12.2 μmol/L). Her plasma methionine level was 20 μmol/L (normal 16‐34 μmol/L). A gene‐testing panel for intracellular cobalamin metabolism defects identified two pathogenic variants in *MMACHC*: c.271dupA; p.Arg91LysfsX14 and c.389A > G; p.Tyr130Cys (Figure 1).

Sibling 3 is the 26‐year‐old sister of the proband (Figure 1 **)**. She was on a regular diet. She had not experienced any acute or chronic neuropsychiatric symptoms or thromboembolic complications. Her physical examination was unremarkable. Her brain and spine MRI imaging were normal. Her biochemical evaluations were notable for normal serum vitamin B12 (1189 pg/mL; normal 232‐1245 pg/mL), significantly elevated serum MMA (28.5 μmol/L; normal 0.045‐0.325 μmol/L) and tHcy levels (162.98 μmol/L; normal 0‐12.2 μmol/L). She was confirmed to be a compound heterozygote for the same two pathogenic variants in *MMACHC* as the proband by gene testing (Figure 1).

Sibling 1 is a 29‐year‐old older sister of the proband (Figure 1 **)**. Her medical history included a hearing impairment of uncertain etiology, unilateral renal hypoplasia, hypertension, and chronic renal insufficiency (stage 3a). She was on a regular diet. She had not experienced any acute or chronic neuropsychiatric symptoms or thromboembolic events. Her physical examination was unremarkable. Her brain and spine MRI imaging were normal. Her biochemical evaluation was notable for normal serum vitamin B12 level (705 pg/mL; normal 232‐1245 pg/mL), elevated serum MMA (60.7 μmol/L; normal 0.045‐0.325 μmol/L) and tHcy (215 μmol/L; normal 0‐12.2 μmol/L). She was confirmed to be a compound heterozygote for the same two pathogenic variants in *MMACHC* as her two siblings by gene testing (Figure 1 **)**.

**Figure 3:**
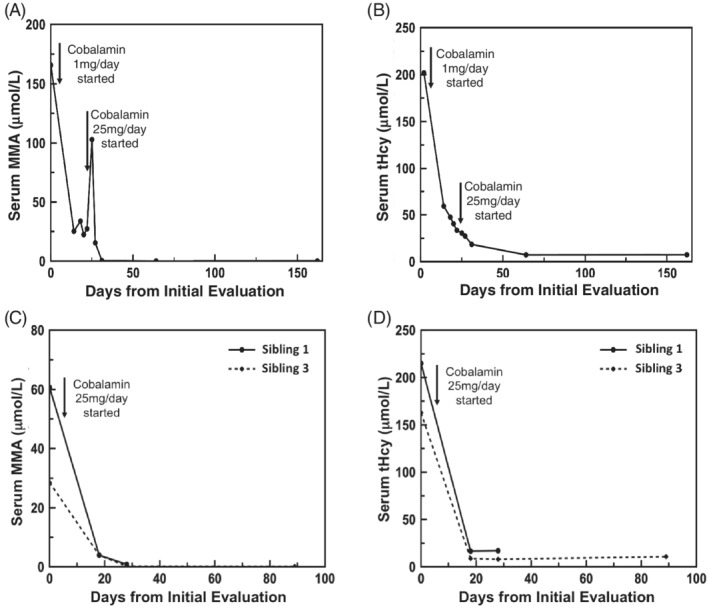
Serum Total Homocysteine (tHcy) and Methylmalonic Acid (MMA) Levels in Three Patients with cblC deficiency in Response to High‐dose Hydroxocobalamin Injection. Panel A, serum MMA levels of the proband patient who received hydroxocobalamin injections of the two indicated doses over 6 months. Note a significant reduction of MMA levels on 1 mg daily injection and a normalization of MMA levels on 25 mg daily injection; note the proband patient switched from a strict vegetarian diet to a regular protein diet around days 24‐25. Panel B, serum tHcy levels of the proband who received hydroxocobalamin injection of the two indicated doses over 6 months. Note a significant reduction of tHcy levels on 1 mg daily injection and a normalization of tHcy levels on 25 mg daily injection. Panel C, serum MMA levels of siblings 1 and 3 who received hydroxocobalamin injections at the indicated doses over 1 and 3 months, respectively. Note a normalization of MMA levels for both patients on this regimen. Panel D, Serum tHcy levels of siblings 1 and 3 who received hydroxocobalamin injection at the indicated doses over 1 and 3 months, respectively

The proband was started with hydroxocobalamin injection at 1 mg/day (0.02 mg/kg/day), betaine at 250 mg/kg/day, and folic acid at 1 mg/day, which resulted in rapid reduction in serum tHcy and MMA levels during the first three weeks (Figure 3A,B). After this initial period, however, her serum tHcy and MMA levels plateaued above the normal ranges. The hydroxocobalamin injection dose was then increased to 25 mg daily (0.5 mg/kg/day) to maximize therapeutic effects. Metanx (L‐methylfolate, pyridoxal‐5′‐phosphate, and methylcobalamin) was also added to the treatment regimen. Her serum tHcy and MMA levels rapidly normalized in about 3 weeks and remained within normal range at 6 months on this regimen, (Figure 3A,B). Her plasma methionine level increased to 31 μmol/L (normal: 16‐34 μmol/L) after 2 months of treatment. Her serum vitamin B12 levels were significantly elevated to the range of 1 × 10^6^ pg/mL (normal, 232‐1,245 pg/mL).

After less than 2‐months of high‐dose hydroxocobalamin injection, her psychosis was completely resolved. Her urinary function was normalized. The patient was able to ambulate using a walker. Her neurologic examination was notable for retained strength, lower extremity spasticity, few beats of clonus at the bilateral patellae and ankles, and normalized vibratory and proprioceptive sensory functions. A repeat brain MRI at 3 months showed much reduced periventricular white matter lesion burden and less conspicuous T2 hyperintense signals in the basal ganglia (Figure 2C). A repeat spine MRI showed similar dorsal column involvement but with a much smaller cross‐section extending from C2‐T10 levels (Figure 2D). The patient's neurological status continued to improve with high‐dose hydroxocobalamin treatment and intensive physical rehabilitation. After 4‐months of treatment, she was able to ambulate unassisted; however, her lower extremity spasticity persisted. Neurocognitive testing showed IQ within the normal range. Her high‐dose injections are being continued at 25 mg/day until she has achieved a full neurologic recovery or has shown no further improvement for several months.

Siblings 1 and 3 were treated with the same hydroxocobalamin injection regimen at 25 mg daily, together with oral supplementation with betaine, folic acid, and Metanx. Both patients tolerated this regimen without any complications. Their abnormal serum MMA and tHcy levels were corrected within 3 weeks of treatment (Figure 3C,D). Their hydroxocobalamin injections were successfully spaced out to 25 mg weekly and their serum MMA and tHcy levels remained in good metabolic control (Figure 3C,D).

## 3 DISCUSSION

We report three adult siblings with biochemically and molecularly confirmed *cblC* deficiency. The proband presented with psychiatric disturbances, progressive neurological deterioration, and thromboembolic complications. Sibling 1 had a chronic kidney disease, while sibling 3 was seemingly asymptomatic. All three siblings share the same pathogenic variants in *MMACHC* and similarly elevated tHcy and MMA levels. However, their differing clinical manifestations suggest that other genetic and/or environmental risk factors played a role. Late‐onset *cblC* deficiency in adults often poses a diagnostic challenge, resulting in delayed diagnosis and treatment. Initial evaluations of adults with new onset of neuropsychiatric symptoms often include serum vitamin B12 levels. However, serum vitamin B12 level are often normal in *cblC*, and serum tHcy and MMA levels are not routinely ordered to evaluate for an intracellular vitamin B12 metabolism defect.^16^ All three siblings in this family were found to have normal vitamin B12 levels at diagnosis. Our report highlights the importance of evaluating adults with neuropsychiatric presentations, thromboembolic, and/or renal complications for potential genetic defects of intracellular cobalamin metabolism. In the presence of normal vitamin B12 levels, it is critical to specifically evaluate serum tHcy and MMA levels in these patients.

The proband (sibling 2) initially presented with prominent neuropsychiatric deterioration. Her MRIs identified spinal cord and white matter lesions, which are commonly seen in patients with severe cobalamin deficiency. Intriguingly, her brain MRIs also identified significant basal ganglia involvement, which has been described in patients with the early‐onset form (6 of 49 patients in one study cohort)^17^ but rarely in adults with the late‐onset form Reference 18. However, she did not experience extrapyramidal symptoms localizing to this finding, and its significance remains unclear. Treatment with high‐dose hydroxocobalamin injections, betaine, and folic acid resulted in complete resolution of psychosis, as well as significant improvement in mood symptoms and neurological function in the proband. When comparing her neuroimaging before and after treatment, there appears to have a significant improvement in lesions in the basal ganglia, spinal cord, and periventricular white matter of brain. These findings support previous observations that encephalopathy and brain and spinal cord lesions in patients with *cblC* deficiency are potentially reversible.^19^ Therefore, timely confirmation of the diagnosis and implementation of effective treatment are critical to slow and reverse disease progression, reducing the risk of developing serious complications and maximizing neuropsychiatric recovery for patients with *cblC*.

High‐dose parenteral administration of hydroxocobalamin is generally considered safe and is FDA approved for the treatment of cyanide poisoning (5 g IV infusion).^20^, ^21^ There is limited information about the optimal frequency and dosing of hydroxocobalamin in *cblC* and historically 1 mg/day has been used throughout the lifespan and is recommended by current guidelines.^10^, ^15^ In recent years, several studies evaluated escalating doses of hydroxocobalamin injection in children with *cblC* deficiency in an attempt to improve biochemical and clinical outcomes. In one study, two young children aged 4 and 12 years with *cblC* deficiency and severe renal involvement were treated with hydroxocobalamin injection up to 5 mg daily, together with betaine.^14^ The treatment regimen achieved a significant reduction of serum MMA and tHcy levels, with improvement of hematuria, proteinuria, and renal insufficiency. In the next study, a 13‐year‐old patient with *cblC* deficiency, homozygous for p.Arg91LysfsX14 in *MMACHC*, was given escalating doses of hydroxocobalamin injection from 1 mg, three times per week; to 10 mg, three times per week; to 10 mg daily; to 20 mg daily.^12^ A dose‐dependent metabolic response was observed with 80% reduction of serum MMA, 55% reduction of tHcy, and 2‐fold increase in methionine levels at 20 mg daily. No adverse effects were reported. In the third study, five children with early‐onset *cblC* deficiency received escalating doses of hydroxocobalamin injection starting from 16 days to 5 months of age.^13^ Four of the five patients were homozygotes for p.Arg91LysfsX14, and one was a compound heterozygote for p.Arg91LysfsX14 and p.Tyr205X. Initial hydroxocobalamin injection doses were individualized and varied from 1 to 10 mg every 72 hours. Escalating dosing regimens were also individualized and varied from 5 mg every 72 hours to 15 mg every 48 hours. During the 18 to30‐months of treatment, two patients showed favorable metabolic responses with significant reduction of tHcy and MMA levels, two patients showed variable reduction of tHcy and/or MMA levels, and one patient showed no reduction in either tHcy or MMA level.^13^ Clinically, two patients showed improvement in their behaviors, communication, and socialization skills while the other three patients did not.^13^ In a most recent report, three young children with early onset *cblC* deficiency caused by homozygous variants, p.Arg91LysfsX14, were treated with dose intensification of parenteral hydroxocobalamin between 2.4 and 4.1 mg/kg/day.^22^ Results indicated good biochemical responses and positive effects in favor of clinical efficacy with high‐dose regimens. No adverse effects were observed.

An optimal dosing regimen for parenteral hydroxocobalamin in adults with *cblC* deficiency has not been systematically evaluated.^10^, ^15^ For the proband, the standard dose injection at 1 mg daily resulted in a significant reduction for serum tHcy and MMA levels, which appeared to have plateaued after 3 weeks of treatment. High‐dose injection at 25 mg daily (~0.5 mg/kg/day) resulted in normalization of tHcy and MMA levels, resolution of psychotic symptoms, significant recovery of neurological manifestations, and improvement of brain and spinal cord lesions on neuroimaging. For the two siblings, 25 mg daily hydroxocobalamin injection resulted in a rapid biochemical correction. After this period, hydroxocobalamin injection was successfully spaced out to a 25 mg weekly injection, which achieved in a good and sustainable metabolic control. No adverse effect was reported with this regimen. Our study showed that 25 mg/day of hydroxocobalamin injection regimen achieved rapid and satisfactory biochemical control compared to the standard 1 mg/day injection in the proband. Because clinical symptoms and organ damage in patients with *cblC* deficiency are potentially reversible and high‐dose hydroxocobalamin injection is associated with minimal side effects, it would be reasonable to consider a high‐dose regimen to achieve a rapid biochemical control and maximal clinical improvement, at least during the acute phase. Once biochemical control is achieved, we showed that injections could be spaced to weekly (and potentially longer) to sustain satisfactory biochemical control in two siblings with identical genotypes. Systematic studies with long‐term follow up of large cohorts of patients with clearly defined genotypes will be needed to identify the optimal dosing regimens for patients with late‐onset *cblC* deficiency.

Patients with *cblC* deficiency show considerable differences in their biochemical and clinical responses to hydroxocobalamin injection, which may in part depend on age/weight and genotype.^12^, ^13^ Our patients are compound heterozygotes for two pathogenic variants in *MMACHC*, p.Arg91LysfsX14 and p.Tyr130Cys.^1^, ^23^ p.Arg91LysfsX14 accounts for 42% of all pathogenic alleles in a large cohort of patients with *cblC* deficiency^23^ and is predicted to cause premature termination before the vitamin B12 binding domain of MMACHC.^1^ Patients homozygous for p.Arg91LysfsX14 often present with severe, early‐onset disease.^8^ p.Tyr130 is located within the vitamin B12 binding domain.^1^ Two neighboring amino acid residues of p.Tyr130 were found to interact directly with vitamin B12 based on a crystal structure.^24^ p.Tyr130Cys was observed in two unrelated patients in a study of mutations in 118 patients with *cblC* deficiency.^23^ Both patients were shown to be compound heterozygotes for p.Arg91LysfsX14 and p.Tyr130Cys, with one symptomatic starting at one year of age.^23^ Additionally, a recent study of seven patients with late‐onset *cblC* deficiency and renal disease identified arteriolar and glomerular thrombotic microangiopathy as a shared renal pathology^25^ Two of the seven patients, aged 20 and 26 years, were found to be compound heterozygous for p.Arg91LysfsX14 and p.Tyr130Cys in MMACHC.^25^ Six patients in this cohort were treated with parenteral hydroxocobalamin and found to have good biochemical responses and clinical improvement.^25^ One sibling in this current family was noted to have chronic renal disease, stage 3a of unknown cause. It is encouraging that she responded well to high‐dose hydroxocobalamin injection with normalization of her serum MMA levels. Her serum tHcy levels remained slightly above the normal range in recent measurements. Close monitoring of her renal functions on hydroxocobalamin injection is needed. Our current study demonstrates the importance of evaluating intracellular cobalamin metabolism in adults with neuropsychiatric symptoms and/or thromboembolic events, and once confirmed, screening asymptomatic at risk relatives.

## CONFLICT OF INTEREST

The authors declare that they have no conflict of interest.

## AUTHOR CONTRIBUTIONS

T.H., A.Y.K, J.T.O., M.A.A., and J.M.C contributed to the collection, analyses and interpretation of data, and drafting the article. J.L.S., I.M., C.P.V., M.G‐A, and T.W. contributed to conception and design, analyses and interpretation of data, and drafting the article or revising it critically for important intellectual content. All authors read and approved the final manuscript.

## DETAILS OF ETHICS APPROVAL

This study is conducted in accordance with the ethical standards of the responsible conduct on human subject research (institutional and national) and with the Helsinki Declaration of 1975, as revised in 2000. The patients were evaluated, in part, under protocol 04‐HG‐0127 “Clinical and Basic Investigations of Methylmalonic Acidemia and Related Disorders” (http://clinicaltrials.gov identifier: NCT00078078). This study was approved by the National Human Genome Research Institute (NHGRI) Institutional Review Board and the research adhered to the tenets of the Declaration of Helsinki. The work is Health Insurance Portability and Accountability Act (HIPAA)‐compliant, and informed consent from the patients was obtained.

## A PATIENT CONSENT STATEMENT

The authors obtained patient consent to use protected health information presented in this report.
